# Identification of the shared gene MXD3 signatures and biological mechanism in patients with hip pain and prostate cancer

**DOI:** 10.1097/MD.0000000000039592

**Published:** 2024-09-13

**Authors:** Liang Huang, Yu Xie, Shusuan Jiang, Binbin Gong, Yao Feng, Hong Shan

**Affiliations:** a Department of Urology, The Affiliated Cancer Hospital of Xiangya School of Medicine, Central South University, Hunan Cancer Hospital, Changsha, Hunan, China; b Department of Urology, The First Affiliated Hospital of Nanchang University, Nanchang, Jiangxi, China; c Department of Stomatology, The Second Xiangya Hospital, Central South University, Changsha, Hunan, China; d Department of Emergency Medicine, The Affiliated Changsha Central Hospital, Hengyang Medical School, University of South China, Changsha, Hunan, China.

**Keywords:** crosstalk genes, hip pain, immune infiltration, MXD3, MXI1, prostate cancer

## Abstract

Prostate cancer (PRAD) is recognized as having a significant effect on systemic illnesses. This study examined possible immune cells, metabolic pathways, and genes that may explain the interaction between PRAD and hip pain. We used information retrieved from the Cancer Genome Atlas and the Gene Expression Omnibus databases. To find common genes, we utilized differential expression analysis and weighted gene co-expression network analysis. The genes that were shared were subjected to pathway enrichment studies using Gene Ontology and the Kyoto Encyclopedia of Genes and Genomes. Additionally, hub genes were analyzed using LASSO regression, and a receiver operating characteristic curve was generated based on the screening outcomes. The genes for the nodes were chosen in a protein–protein interaction network that was built. Single-sample gene-set enrichment analysis was performed to identify the differentially expressed genes. Immunohistochemistry staining confirmed hub gene expression, and single-sample gene-set enrichment analysis assessed immune cell infiltration. We concluded by comparing MAX dimerization protein 3 (MXD3) and MAX interactor 1 (MXI1) expression in tumor tissues using Uniform Manifold Approximation and Projection and violin plots in the Tumor lmmune Single-cell Hub database. After analyzing the intersection of the differentially expressed genes and weighted gene co-expression network analysis-significant module genes, we determined that MXD3 was the best shared diagnostic biomarker for PRAD and hip pain. One potential predictor of PRAD development was the MXI1 node gene, which was found in the protein–protein interaction network. The analyses revealed that MXD3 had a relatively positive correlation with neutrophil and T-helper cell infiltration levels, whereas MXI1 had a negative correlation with mast and Tgd cell levels. Tumors had lower levels of MXI1 expression and higher levels of MXD3 expression compared to normal tissues. Endothelial cells, induced pluripotent stem cells, and smooth muscle cells were all found to express MXI1. This is the first study to investigate the close genetic link between hip pain and PRAD using bioinformatics technologies. The 2 most significant genes involved in crosstalk between PRAD and hip pain were MXD3 and MXI1. The immunological responses triggered by T cells, mast cells, and neutrophils may be crucial in the relationship between PRAD and hip pain.

## 1. Introduction

Globally, prostate cancer (PRAD) is the most common non-skin cancer in males, accounting for about 1.6 million cases and 366,000 fatalities annually.^[1]^ For those afflicted with this disease, it presents serious medical risks.^[2]^ Although there is a good likelihood of long-term survival for patients with localized PRAD, metastatic PRAD is still mostly incurable, even after extensive multimodal therapy.^[3]^ Because there are no effective therapy alternatives that can produce sustained responses in the context of high tumor heterogeneity at the genetic and cellular levels, the advanced stages of this illness are deadly.^[4]^

Pain in the hip is a common and incapacitating symptom among people 60 years of age or older.^[5,6]^ The incidence of PRAD and death are also connected with growing older, and hip pain may have rare causes, such as infection, aortoiliac insufficiency, or bone metastases.^[7]^ However, these are not the only causes of hip pain. It is interesting to note that PRAD is the most common type of cancer that spreads to the bone, typically affecting the hips, ribs, spine, or pelvis.^[8,9]^ In the later stages of the disease, patients frequently develop bone metastases, which typically manifest as painful bone pain, pain in the nerve roots, neurological abnormalities, or dysfunction in the bladder. In more advanced stages of metastasis, the cancer may also spread to other organs of the body, such as the lungs, adrenal glands, liver, and pleura.^[10,11]^ Bone metastasis, despite its rarity, has the potential to induce persistent and incapacitating hip pain.^[5]^ This highlights the need to identify potential molecular targets for the diagnosis and therapy of both post-traumatic arthritis of the PRAD and hip pain.

Additional research is needed regarding the connection between hip pain and PRAD, particularly with regard to the cellular and molecular pathways involved. In light of significant advancements in microarray and high-throughput sequencing technologies, procedures in the field of bioinformatics are frequently utilized to investigate the crosstalk that occurs between diseases. The purpose of this study was to gain a more in-depth understanding of the pathophysiological processes that may be linked to hip pain and PRAD. To do this, we utilized bioinformatics techniques to examine the potential genes involved in the crosstalk between hip pain and PRAD. We also evaluated the interaction between these potential crosstalk genes and the immune cells that infiltrate the immune system.

## 2. Materials and methods

### 2.1. Determination of differentially expressed genes (DEGs)

The R (4.0.4) software was utilized for the normalization and processing of the initial expression matrix. For the purpose of screening DEGs from the GSE124272 and the Cancer Genome Atlas (TCGA) datasets, the “limma” R program was utilized. Screening was performed to determine whether the DEGs were suitable for datasets that had an adjusted *P*-value of <.05 and an absolute value of the logarithmic fold change of at least 1. The R programming language was utilized to produce a heatmap and a volcano map for differential gene clustering.

### 2.2. Weighted gene co-expression network analysis (WGCNA) network architecture and identification of modules

A bioinformatics research technique called WGCNA was used to characterize patterns of gene association among various samples (hip pain in GSE150408 and PRAD in TCGA). It can examine the relationship between modules and particular features or phenotypes and cluster genes with comparable patterns of expression. The co-expression network was built using the WGCNA R software package. The WGCNA contained genes with an adjusted *P*-value of <.05. The standard R function “Hculst” was used to perform hierarchical clustering to determine whether any clear outliers existed. The suitable soft thresholding power b was chosen using the “pickSoft Threshold” function to align the gene expression relationship with the scale-free network. Third, the gene expression similarity matrix was transformed into an adjacency matrix based on the soft-thresholding parameter b using the “adjacency” function. Fourth, to reduce the impact of noise and spurious associations, the adjacency matrix acquired in the preceding phase was converted into a topological overlap matrix. Ultimately, modules were identified using hierarchical clustering and the dynamic tree cut function, and the relationship between modules and patient clinical features (*P* < .05) was examined using Pearson correlation.

### 2.3. Identification of shared DEGs and pathway enrichment

Through Venn diagrams, a combined analysis of the genes screened using WGCNA and of DEGs was carried out. Genes that overlapped were regarded as common core genes, and they were retrieved to conduct additional functional enrichment analysis. To perform pathway enrichment studies, the “enrichplot” and “ggplot2” packages in R were utilized. These analyses were performed using Gene Ontology (GO) and the Kyoto Encyclopedia of Genes and Genomes (KEGG). Statistical significance was established at a level of *P* < .05.

### 2.4. Building nomograms and the receiver operating characteristics (ROC) curve

To assess each putative biomarker’s diagnostic potential, its expression was compared, and a ROC curve was produced in GSE124272 and TCGA. The diagnostic value was then estimated using the area under the receiver operator curve (AUC), which was computed and used with a 95% confidence interval. To prevent bias, the validation sets were the GSE150408 and GSE70768 datasets. Using the R package, a nomogram was constructed only for candidates whose AUC was more than 0.5 in both the test and validation sets. The nomogram’s diagnostic efficacy was confirmed by calculating the AUC.

### 2.5. Building a network of protein–protein interactions (PPIs)

To create the PPI network with a minimum interaction score of 0.400 and to elucidate the relationships between DEGs, the STRING database (v11.5) (www.string-db.org) was utilized. Genes that did not interact with one another were eliminated in the first round of selection. Cytoscape was used to visualize the TSV data downloaded from the database. The degree algorithm in the Cytoscape CytoHubba plug-in was used to select the top 30 DEGs, which were then displayed as node genes for the second round of selection.

### 2.6. Examination of immune cells

We used CIBERSORT analysis to determine the level of immune cell infiltration of 22 immunocytes in the neuropathic pain group.^[12]^ This was done to study the role that immune cells play in the diagnosis and treatment of PRAD in TCGA and hip pain in GSE124272.

### 2.7. Staining via immunohistochemistry

Additionally, MAX dimerization protein 3 (MXD3) and MAX interactor 1 (MXI1) protein expression immunohistochemistry pictures (n = 11 each) were acquired from PRAD and para-carcinoma tissues. Individual sample sizes and human cancers were gathered from a cancer hospital affiliated with Xiangya Medical College, Central South University (Table S1, Supplemental Digital Content, http://links.lww.com/MD/N545). Using 3% hydrogen peroxide and 5% goat serum, ATMA slides were inhibited. The slides were first incubated with primary antibodies for an entire night at 4 °C. They were then left to be incubated for 30 minutes at room temperature with an enhancer and a secondary antibody. An HRP-conjugated compact polymer system was used for detection. As the chromogen, DAB was employed. Hematoxylin was used as a counterstain, and DPX was used to mount the tissues. Primary antibodies included the rabbit polyclonal antibody against MXI1 (ER61812, HUBIO, China, 1:200) and the recombinant rabbit monoclonal antibody against MAD3 (ET7109-76, HUBIO, China, 1:200). Two pathologists who were blind to the provenance of the clinical samples evaluated and rated the immunohistochemistry staining results. We employed a semiquantitative integration method to analyze staining intensity using ImageJ software (National Institutes of Health, Bethesda, MD) software.

### 2.8. Single-sample gene-set enrichment analysis (ssGSEA)

ssGSEA was performed to identify the differentially expressed gene sets between the low- and high-risk cohorts. The enrichment score represents the degree of absolute enrichment of a gene set in each sample within a certain dataset. Using the GSVA package and its ssGSEA method (http://www.bioconductor.org), the enrichment scores in each sample were calculated as the normalized differences in the empirical cumulative distribution functions of gene expression ranks inside and outside the gene set.^[13]^ The most significantly differentially expressed gene sets (*P*-value < .001) were generated for further analysis.

### 2.9. Analysis of data from a single cell

The TISCH database provided us with the raw data for GSE193337 of PRAD, which we downloaded. Following the completion of a series of dimensionality reduction clustering and corresponding cell annotations, we annotated each cell population into distinct cells and then used UMAP and violin plots to demonstrate the expression of the gene in each cell type.

## 3. Results

### 3.1. Identification of DEGs and pathway enrichment

**Figure 1.**
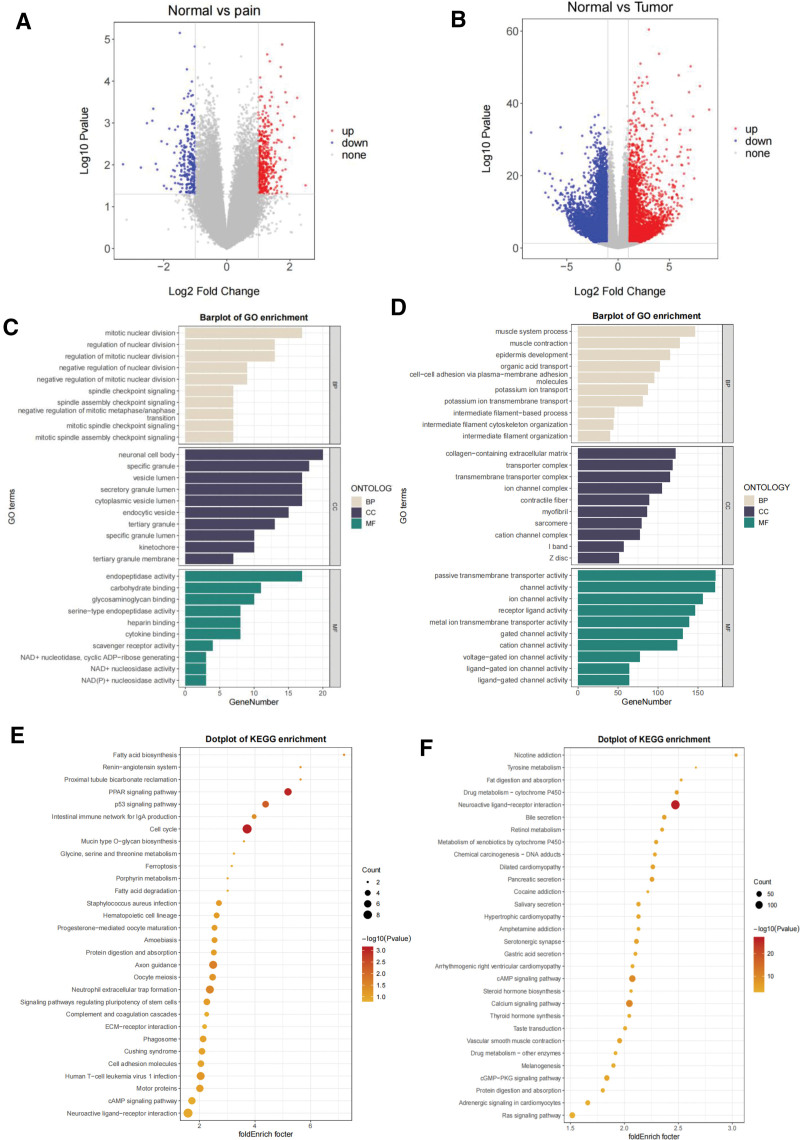
Identification of differentially expressed genes. (A) A volcano map in GSE124272 with DEGs. (B) A TCGA volcano graphic of PRAD’s DEGs. (C and E) DEG functional enrichment analysis in GSE124272. The DEGs’ KEGG pathway (E) and GO analysis (C) enrichment analyses. (D and F) Functional enrichment analyses of PRAD’s DEGs in TCGA. The DEGs’ KEGG pathway (F) and GO analysis (D) enrichment analyses. DEGs = differentially expressed genes, GO = Gene Ontology, KEGG = the Kyoto Encyclopedia of Genes and Genomes, pain = hip pain; tumor, PRAD = prostate cancer; TCGA = the Cancer Genome Atlas datasets.

Out of the 523 DEGs that were found in the hip pain dataset GSE124272, 317 DEGs were upregulated and 206 DEGs were downregulated (Fig. 1A). A heatmap of the DEGs is shown in Figure S1, Supplemental Digital Content, http://links.lww.com/MD/N545 Out of the 9885 DEGs found in the PRAD dataset of TCGA, 4215 DEGs were upregulated and 5670 DEGs were downregulated (Fig. 1B). A heatmap of the DEGs is shown in Figure S2, Supplemental Digital Content, http://links.lww.com/MD/N545. The functional enrichment analyses of the DEGs were performed with GO and KEGG annotations. The DEGs were primarily enriched in numerous categories, according to the results of the GO enrichment analysis performed on the GSE124272 database (Fig. 1C). These categories included the following: (1) mitotic nuclear division, (2) neuronal cell body, and (3) endopeptidase activity. According to the results of the GO enrichment analysis performed on TCGA database, DEGs were primarily enriched in a few categories, including the following: (1) muscle system process, (2) collagen-containing extracellular matrix, and (3) passive transmembrane transporter activity (Fig. 1D). The KEGG pathway enrichment analysis showed that these genes were largely prominent in the PPAR signaling pathway, the cell cycle and axon guidance in GSE124272, and the neuroactive ligand–receptor interaction in TCGA (Fig. 1E and F). Based on these findings, it appears that overlapping DEGs are involved in issues related to hip pain and PRAD.

### 3.2. WGCNA network architecture and identification of modules

**Figure 2.**
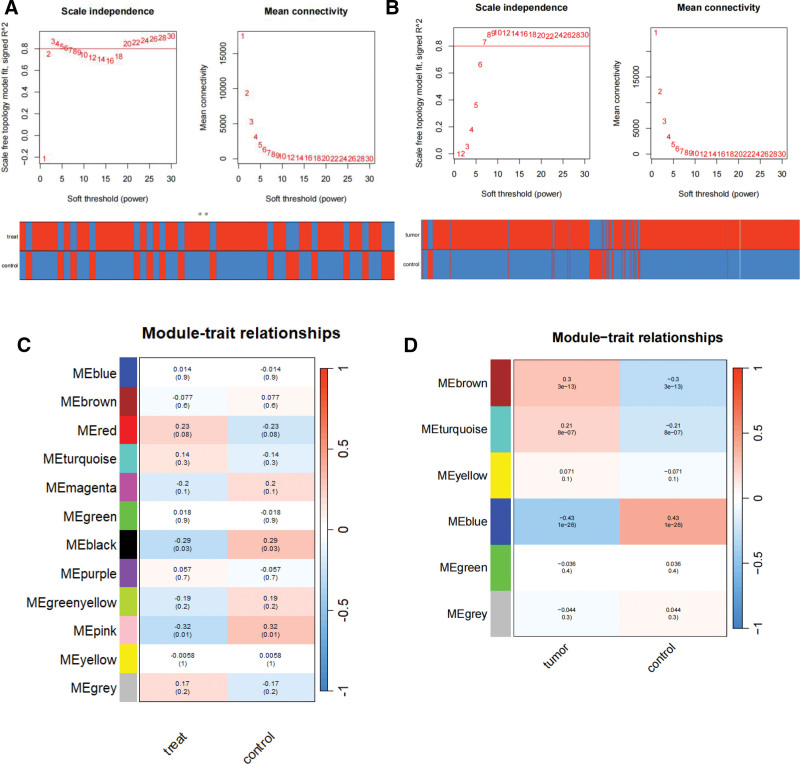
Coexpression analysis for differentially expressed genes. (A) Pick soft threshold and trait heatmap in GSE150408. (B) Pick soft threshold and trait heatmap of PRAD in TCGA. (C) Heatmap of the module-trait relationships in GSE150408. (D) Heatmap of the module-trait relationships of PRAD in TCGA. TCGA = the Cancer Genome Atlas datasets; Treat = traditional Chinese medicine treatment; Tumor, PRAD = prostate cancer.

Prior research has examined the robust association between hip discomfort and PRAD. The hub genes of this stemness-related cluster were found using WGCNA. To ensure the creation of a scale-free network, a power *β* of 6 was determined to be the ideal soft threshold for GSE150408 and TCGA (Fig. 2A and B). In the co-expression network built by GSE150408, we obtained 12 modules, while in the network built by TCGA, we obtained 4 modules. Our goal was to find genes linked to the development of diseases by examining the relationship between modules and clinical characteristics. In the GSE150408 database in Figure 2C, the black (*r* = −0.29, *P* < .05) and pink (*r* = −0.32, *P* < .05) modules showed the strongest negative link for hip pain, whereas the red module showed the strongest positive relation (*R* = 0.23, *P* < .10). In TCGA database of Figure 2D, the blue module had the largest negative association (*r* = −0.43, *P* < .01), and the turquoise and brown modules had the strongest positive correlations (*R* = 0.21, *P* < .01) for PRAD.

### 3.3. Identification of shared genes

**Figure 3.**
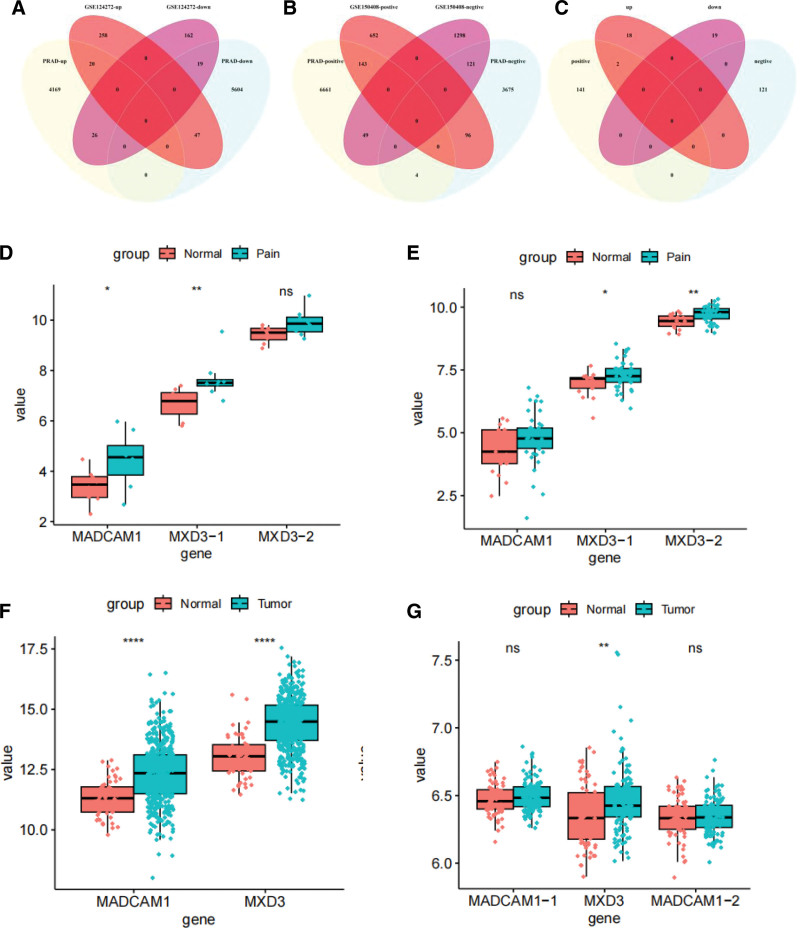
Identification of the shared genes. (A) Venn diagram showing an overlap of 39 DEGs between hip pain and PRAD. (B) Venn diagram showing 264 genes overlap in hip pain and PRAD modules. (C) Venn diagram showing that 2 score genes were crossed and overlapped between the genes screened by DEGs and WGCNA. (D and E) Expression of MADCAM1, MXD3 in GSE124272 (D) and GSE150408 (E). (F and G) Expression of MADCAM1, MXD3 in TCGA (F) and GSE70768 (G). DEG = differentially expressed gene; MXD3 = MAX dimerization protein 3; Pain = hip pain; Tumor, PRAD = prostate adenocarcinoma; WGCNA = weighted gene co-expression network analysis.

An example dendrogram of WCGNA genes with differential expression in GSE150408 and TCGA is presented in Figure S3, Supplemental Digital Content, http://links.lww.com/MD/N545. A Venn diagram was used to show where the GSE124272 and PRAD hub modules intersected, and 39 intersection genes were found (Fig. 3A). A Venn diagram was also used to show where the GSE150408 and PRAD hub modules intersected, and 264 intersection genes were found (Fig. 3B). The potential crosstalk genes between the 2 disorders, MADCAM1 and MXD3-1, were found to cross and overlap between the genes screened by WGCNA and DEGs (Fig. 3C). The expression of MXD3-1 and MADCAM1 was much higher in GSE124272 than in normal tissue (Fig. 3D). The expression of MXD3-2 and MXD3-1 was greater in GSE150408 than in normal tissue (Fig. 3E). In TCGA and GSE70768 databases, MADCAM1 and MXD3 expression was greater than that in the control group (Fig. 3F and G).

### 3.4. Selection of putative common diagnostic genes using the ROC curve

**Figure 4.**
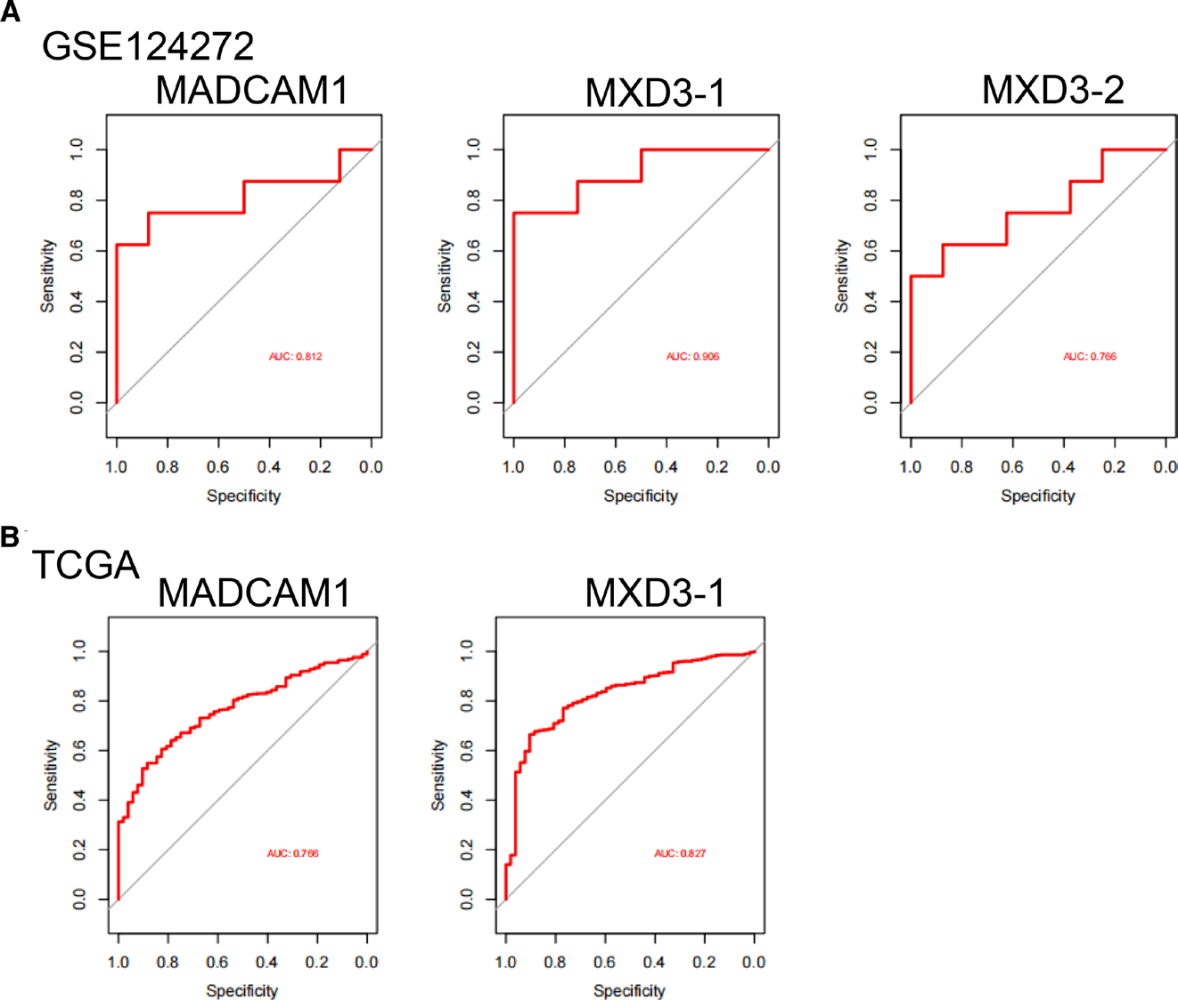
Expression pattern validation and diagnostic value. (A) ROC curve of MADCAM1, MXD3 in GSE124272. (B) ROC curve of the shared diagnostic genes in TCGA. MXD3 = MAX dimerization protein 3, ROC = the receiver operating characteristics, TCGA = the Cancer Genome Atlas datasets.

We assessed each proposed biomarker’s sensitivity and specificity. The diagnostic values of MXD3-1 (AUC = 0.906) and MADCAM1 (AUC = 0.812) in the GSE124272 dataset (Fig. 4A) were good. We also assessed MXD3-1 (AUC = 0.827) and MADCAM1 (AUC = 0.766), both of which had good sensitivity in TCGA datasets (Fig.4B). Within the GSE150408 dataset (Fig. S5A, Supplemental Digital Content, http://links.lww.com/MD/N545), the following MXD3 biomarkers still had strong diagnostic values: AUC values of 0.676 for MXD3-1 and 0.751 for MXD3-2. The diagnostic efficacy of MXD3 and MADCAM1 was subsequently externally validated in GSE70768, and all results demonstrated the predictive performance of MXD3 (AUC = 0.628) (Fig. S5B, Supplemental Digital Content, http://links.lww.com/MD/N545).

### 3.5. Associations between MXD3 and shared genes with the PPI network

**Figure 5.**
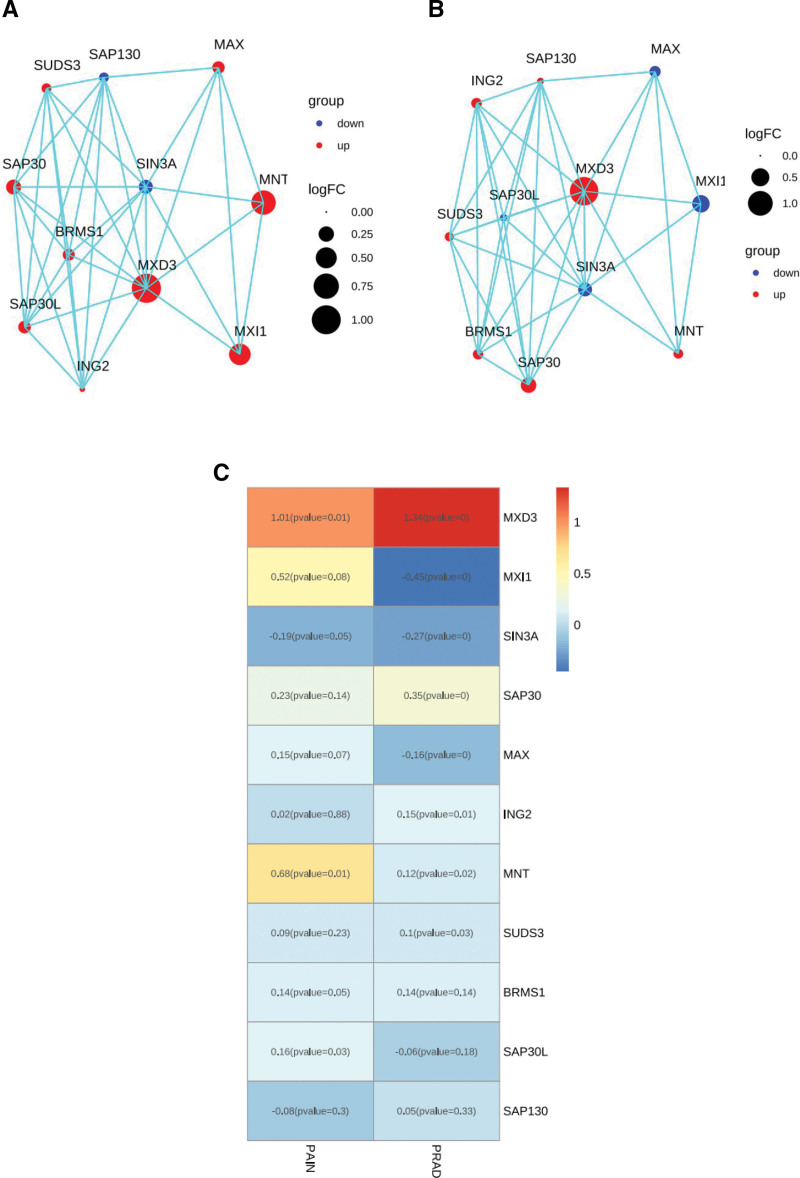
Protein–protein interaction (PPI) network construction. (A) The top 10 DEGs’ connection with MXD3 in hip pain is displayed by the PPI network. (B) The top 10 DEGs’ connection with MXD3 in PARD is displayed via the PPI network. (C) Heatmap showing the best shared 10 DEGs for PARD and hip pain. DEGs = differentially expressed genes; MXD3 = MAX dimerization protein 3; PRAD = prostate cancer.

According to the PPI network, 10 distinct genes (MXI1, MNT, MAX, SAP130, SUDS3, SAP30, SAP30L, ING2, BRMS1, and SIN3A) had a significant relationship with MXD3 in the hip pain and PARD database (Fig. 5A and B). We also showed that MXD3 was related to MXI1 in hip pain and that MXD3 was related to MXI1 in PRAD (Fig. 5C).

### 3.6. Identification of MXD3-associated biological pathways

To identify MXD3-associated biological pathways, we performed ssGSEA to analyze the GSE150408, GSE124272, TCGA-PRAD, and GSE70768 datasets using the risk score for classification. As shown in Fig. S4C and D, Supplemental Digital Content, http://links.lww.com/MD/N545 a group of pathways that included endopeptidase activity, mitotic nuclear division, muscle system process, neuronal cell body, and passive transmembrane transporter activity was significantly enriched in the high-risk PRAD patients; however, these pathways were found not to be significantly associated with the risk scores of hip pain patients, which were validated through Pearson correlation analysis (Fig. S4A and B, Supplemental Digital Content, http://links.lww.com/MD/N545).

### 3.7. Immune cell infiltration and the link between candidate biomarkers

**Figure 6.**
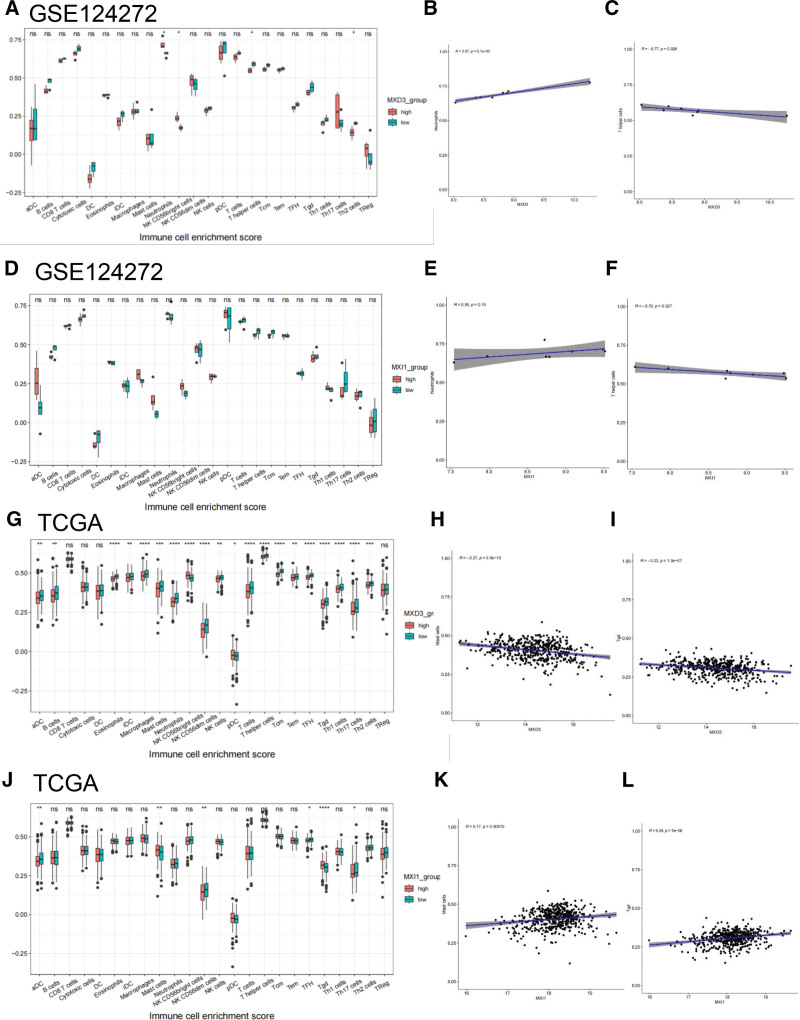
Association of MXD3 and MXI1 gene expression with immune infiltration. In the GSE124272 hip pain sample, the immunological cell infiltration differed between the high-expression and low-expression MXD3 (A) and MXI1 (D) groups. (B, C, E, and F) the examination of association between immune cells and hip pain diagnostic biomarkers. (G and J) The variation in immune cell infiltration between the prostate cancer TCGA high-expression and low-expression MXD3 (G) and MXI1 (J) groups. (H, I, K, and L) the examination of association between immune cell and PRAD diagnostic biomarkers. (J) The variation in immune cell infiltration in the TGCA sample between the MXI1 groups with high and low expression levels. MXD3 = MAX dimerization protein 3, MXI1 = MAX interactor 1, TCGA = the Cancer Genome Atlas datasets.

In hip pain patients in GSE124272, the infiltration level of neutrophils was determined to be higher when MXD3 and MXI1 were highly expressed, while T-helper cells had reverse trends (Fig. 6A and D). The scatter plots shown in Figure 6B (*R* = 0.97, *P* < .01) and 6E (*R* = 0.56, *P* > .05) demonstrate that the expression of MXD3 and MXI1 was positively linked to the presence of neutrophils. On the other hand, the expression of MXD3 (*r* = −0.77, *P* < .05) and MXI1 (*r* = −0.76, *P* < .05) was inversely linked to the presence of T-helper cells (Fig. 6C and F). MXD3 and MXI1 are key regulators of T-helper cells in the development of hip pain.

Regarding PRAD in TCGA, the infiltration levels of aDC, B cells, eosinophils, iDC, macrophages, mast cells, neutrophils, NK CD56bright cells, NK CD56dim cells, pDC, T cells, T-helper cells, TFH, Tgd, Th1 cells, Th17 cells, and Th2 cells were significantly correlated with MXD3 expression (Fig. 6G). The scatter plots are shown in Figure 6H and I, which demonstrate that mast cells (*r* = −0.27, *P* < .01) and Tgd cells (*r* = −0.27, *P* < .01) were negatively linked to MXD3 expression (Fig. 6G–I). There was a significant difference in the levels of infiltration of aDC, mast cells, NK CD56dim cells, Tgd cells, TH 17 cells, and TFH cells between the MXI1-high group and the MXI1-low group. The scatter plots are shown in Figure 6K and L, which demonstrate that mast cells (*R* = 0.17, *P* < .01) and T cells (*R* = 0.24, *P* < .01) had a positive correlation with MXI1 expression. The above results suggest that changes in the mast cells and T cells when MXI1 or MXD3 is downregulated may be key in tumorigenesis.

### 3.8. Validation of the expression of MXD3 and MXI1 in PRAD through the use of clinical specimens

**Figure 7.**
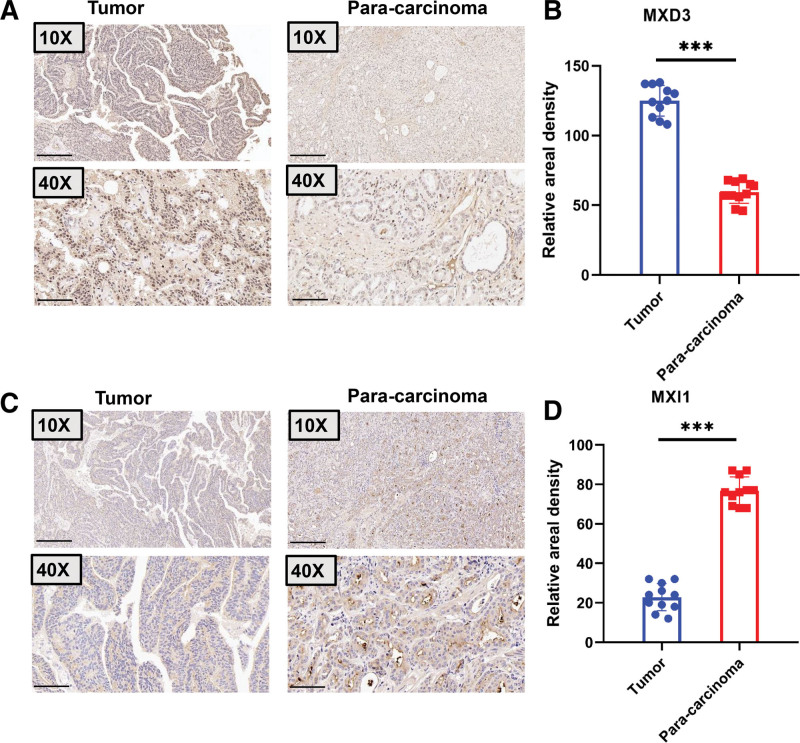
Representative immunohistochemical staining for MXD3 and MXI1 protein in Prostate cancer. (A) MXD3 immunohistochemical staining was done on tumor (n = 11) and para-carcinoma (n = 11) tissues. Scale bar, 50 μm and 200 μm. There are illustrative pictures displayed. (B) MXD3 staining was measured as indicated. ***P < .001. (C) MXI1 immunohistochemical staining was done on tumor (n = 11) and para-carcinoma (n = 11) tissues. Scale bar, 50 μm and 200 μm. There are illustrative pictures displayed. (B) Staining of MXI1 was quantified as shown. ***P < .001. MXD3 = MAX dimerization protein 3, MXI1 = MAX interactor 1.

The expressions of MXD3 and MXI1 were measured using clinical specimens obtained from patients with PRAD. Figure 7A and B show that the expression of MXD3 in the tumors was much higher than that in the para-carcinomatous tissues. As shown in Figure 7C and D, the expression of MXI1 in the tumors was much lower than that in the para-carcinomatous tissues. These findings demonstrate that a possible mechanism in carcinogenesis could be downregulated MXI1 with increased MXD3.

### 3.9. Analysis of single-cell data

**Figure 8.**
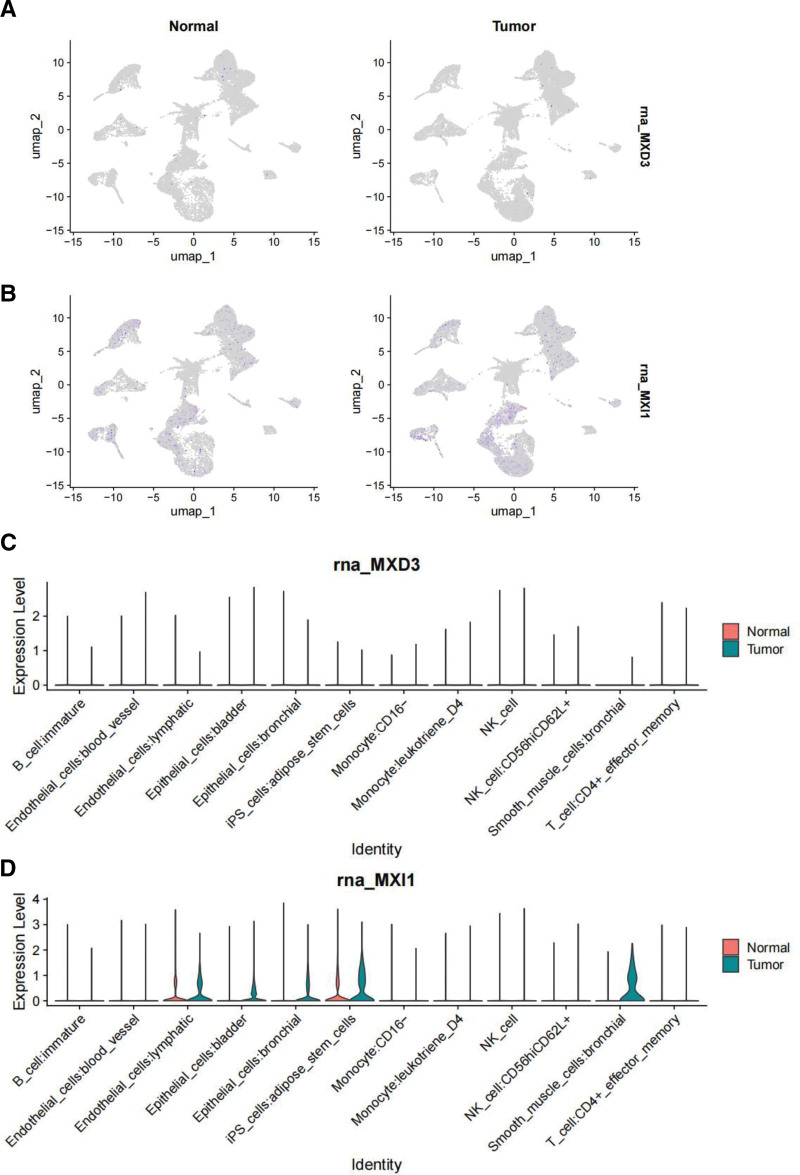
UMAP plots and violin plots. (A and B) UMAP plots of the expression of MXD3 (A) and MXI1 (B) in different identified cell types in prostate cancer and normal tissue from the GSE193337 datasets. (C and D) The GSE193337 datasets’ violin plots of MXD3 and MXI1 expression in several kinds of annotated cells are displayed in (C) and (D), respectively. MXD3 = MAX dimerization protein 3, MXI1 = MAX interactor 1.

From the TISCH database, we extracted raw data from the GSE193337 datasets. The GSE193337 datasets included 12 distinct cell classes annotated using a sequence of downscaling clustering and matching cell annotations. Figure 8 displays the UMAP plots and violin plots of MXD3 and MXI1 expressions in different kinds of annotated cells from the GSE193337 datasets. While MXI1 expression was higher in tumors than in normal tissues (Fig. 8A), there was no discernible change in MXD3 expression in tumors (Fig. 8B). The strong relationship between MXI1 and immunity and carcinogenesis in PRAD was largely supported by the fact that MXI1 was significantly more increased than MXD3 in all types of identified cells, including the close association of MXI1 with lymphatic endothelial cells, adipose stem cells, and iPS cells. Thus, MXI1 and MXD3 mainly regulate immune regulation and tumorigenesis (Fig. 8C and D).

## 4. Discussion

To investigate the shared mechanism between hip pain and PRAD, we merged the transcriptomes of both conditions and utilized WGCNA. This allowed us to identify probable crosstalk genes, shared pathways, and related immune cells. The MXD3, MXI1, and MADCAM1 genes were identified as the most significant crosstalk genes between hip pain and PRAD based on the intersection of WGCNA key module genes and DEGs. These genes may be connected with kinase regulator activity. Ultimately, it was determined that MXD3 and MXI1 were valuable diagnostic markers. They were expressed in all identified cell types, including immune cells, according to the immune infiltration data. This finding partially confirmed the strong relationship between MXD3 and MXI1 and immunity in PRAD.

First, we contrasted the DEGs for PRAD and hip discomfort. On the common genes, KEGG pathway enrichment and GO analysis were carried out. Gao et al used a volcano map to compare the expression of divergent genes in periodontitis and IgAN databases. They discovered that the periodontitis dataset GSE16134 had 232 DEGs in total, 166 of which were upregulated and 66 were downregulated. In IgAN dataset GSE93798, 5730 DEGs were found, of which 1945 were upregulated and 3785 were downregulated.^[14]^ To further clarify the relationship between Alzheimer disease (AD) and ulcerative colitis (UC), Dong et al used the Gene Expression Omnibus database to download the gene expression profiles for AD (GSE5281) and UC (GSE47908). Based on the volcano plots of DEGs from GSE5281, 2639 DEGs (1772 upregulated DEGs and 867 downregulated DEGs) were identified between AD patients and healthy controls. Moreover, 735 DEGs were identified between UC patients and healthy controls based on GSE47908 (479 upregulated DEGs and 256 downregulated DEGs). Finally, 61 co-DEGs with consistent trends were identified in AD and UC.^[15]^ Zhu et al found that endopeptidase was differentially expressed in PRAD cells. The expression in 22RV1 cells was high and that in PC-3 cells was low. The knockout endopeptidase gene significantly inhibited cell proliferation and invasion in 22Rv1 cells, while overexpression in PC-3 cells promoted cell proliferation and invasion. Endopeptidase promoted the activation of the PI3K-AKT signaling pathway in PRAD cells. The results suggest that endopeptidase may be an attractive target for PRAD therapy.^[16]^ Ishikawa et al used the virus as a vector to target the primary somatosensory cortex. The results showed that both pain and itching could activate the calcium signal in the S1 brain region and that 26.3% of S1 neurons could be activated by both pain and itching.^[17]^ Guo et al found liver cancer cells growing on the ECM with different hardnesses and revealed that hardened ECM promoted the release of tumor exosomes. In addition, the exocrine secreted by tumor cells growing on the hardened ECM activated the Notch signal pathway of cells, thus promoting tumor growth.^[18]^ Wang et al found that the PPAR signaling pathway was prevalently and aberrantly activated in colorectal cancer tumors. Blocking the PPAR pathway suppressed the growth and promoted the apoptosis of colorectal cancer organoids in vitro, indicating that aberrant activation of the PPAR signaling pathway plays a critical role in colorectal cancer tumorigenesis.^[19]^ Our findings indicate the correlation between hip discomfort and PRAD, and more research is necessary to determine the potential molecular basis. Furthermore, we carried out GO and KEGG pathway enrichment analyses on common genes and compared DEGs in hip pain and PRAD. This work lays the groundwork for future investigations into the molecular causes of PRAD and hip discomfort.

Weighted gene co-expression network analysis was utilized to determine the genes that served as the hubs of this stemness-related cluster. As the ideal soft threshold for GSE150408 and TCGA, the power *β* of 6 was determined to be the optimal value. Zeng et al employed WGCNA to investigate new hub genes and modules associated with neuropathic pain susceptibility. The WGCNA technique was utilized to build the networks of gene co-expression and to screen for the most pertinent module and for 440 key genes derived from the WGCNA technique that overlapped.^[20]^ The relationship between pancreatic cancer and type 2 diabetes mellitus (T2DM) has long been widely recognized, but the interaction mechanisms remain unknown. Hu et al investigated the shared gene signatures and molecular processes between PC and T2DM. They identified 16 modules in GSE38642 by WGCNA, in which each module was represented by a different color. Based on the Spearman correlation coefficient, a heatmap of module–trait relationships was drawn to assess the relationships between modules. Three modules, “gray,” “darkorange,” and “purple,” had highly positive associations with T2DM, and they were chosen as T2DM-related modules (darkorange module: *R* = 0.43, *P* = 4e−04, genes = 55; purple module: *R* = 0.41, *P* = .001, genes = 107; and cyanmodule: *R* = 0.39, *P* = .002, genes = 290). Additionally, 14 modules were discovered in GSE91035, with the modules “yellow” (*R* = 0.83, *P* = 4e−13, genes = 444) and “darkgreen” (*R* = 0.67, *P* = 2e−07, genes = 1037) being highly positively linked to PC.^[21]^ Some evidence suggests that immune cells actively participate in the pathogenesis of ankylosing spondylitis and inflammatory bowel disease. However, information on which cells are primarily involved in this process and how these cells mobilize, migrate, and interact remains limited. Dong et al imported the PPI network and WGCNA to explore how genes interact with one another. They found that only 49 of 67 upregulated genes were showed, as the function and properties of the remaining 18 genes have not yet been reported in both diseases. Of the 49 genes, only 17 were included in the network, with MyD88 at its core.^[22]^ During the course of this investigation, we were able to acquire a total of 12 modules from the co-expression network built by GSE150408 and 4 modules from the network built by TCGA. We conducted an analysis of the relationship between modules and clinical characteristics to find genes that are connected with the course of diseases or conditions.

MXD3 is a member of the MAX dimerization family of bHLHZ transcription factors, which are critical for controlling the cell cycle and proliferation of cells.^[23,24]^ MXD3 regulates the cell cycle and promotes cell proliferation. The biological activities of MXD3 in PRAD, which have been described,^[25,26]^ served as the impetus for Ma et al’s attempt to investigate the fundamental functions of MXD3. According to their findings, MXD3 encouraged the growth of PRAD cells. In addition, research has demonstrated that MXD3 loss has a significant impact on the stem cell features of PRAD cells.^[27]^ As an additional point of interest, MXD3, which is classified as an onco-immunological biomarker, has been linked to the microenvironment of the tumor, as well as to prognoses, disease stage, and individual responses to various cancer treatments. When all the results are taken into consideration, they indicate that MXD3 plays a significant role in the proliferation and stemness of PRAD, suggesting that it has the potential to be a therapeutic target for patients suffering from PRAD.^[28]^ Wu et al revealed that practically all types of TCGA cancer have unregulated levels of MXD3 mRNA expression. This suggests that the protein possesses the ability to cause cancer through the invasion, metastasis, and progression of the tumor. As a consequence, MXD3 emerges as a promising early biomarker for cancer surveillance and diagnosis.^[29]^ As there are few studies on the connection between MXD3 and hip pain at the moment, additional experiments are required to determine the molecular pathways that connect these 2 illnesses.

Based on the findings, the researchers conclude that 2 fundamental genes MADCAM1 and MXD3-1, intersect and overlap with the genes discovered by WGCNA and DEGs. These genes are possibly involved in the crosstalk that links hip discomfort and PRAD. Particularly noteworthy is the fact that MXD3 is a member of the MAX dimerization family of bHLHZ transcription factors, which are responsible for controlling cell proliferation and the cell cycle. In their pan-cancer investigation of the roles of MXD3, Wu et al reported that MXD3 dysregulation was present in the majority of TCGA cancer types and that this dysregulation was associated with the progression of tumors and their prognoses. Additionally, MXD3 can impede the invasion of T-cells, which in turn promotes immunological evasion of tumors.^[30]^ They discovered that increased MXD3 leads to an increase in the AR signaling pathway, which in turn contributes to the development of hepatocarcinogenesis.^[31]^ A signature that includes MXD3 has been established by Ma et al, and it has demonstrated good performance in prognosis prediction.^[32,33]^ This is despite the limited evidence regarding the biological roles that MXD3 plays in PRAD. They also discovered that MXD3 encourages the proliferation of PRAD cells, although the specific molecular processes responsible for this remain unknown. The information that is now available highlights the significant role that MXD3 plays in the proliferation and stemness of PRAD, indicating that it has the potential to be a promising target for patients with PRAD.^[34,35]^ On the other hand, additional tests are required to shed light on the chemical mechanisms that drive the phenomenon. Considering the high levels of MXD3 expression found in a variety of cancer types, we hypothesize that it may play a substantial role in the disease. In fact, as will be illustrated in the following discussion, our further investigation revealed that MXD3 genetic and epigenetic modifications play a role in the regulation of the tumor immune milieu. To minimize the influence of overfitting and improve the quality of performance indicators, as many samples as possible should be selected for clinical biomarker discovery experiments. In our study, the performance of a biomarker was often assessed using the AUC. The AUC value is between 0 and 1, and the higher the value, the better the overall performance of the test.^[36,37]^ To compare the relationship between flora and the effects in allogeneic fecal microbiota transplantation, Kootte et al used an ROC curve to analyze the diversity of microflora, corresponding to a random result. They defined a critical value of 0.5 and used 0.5 AUC to measure the prediction accuracy of the classification model. In the present study, we found that the diagnostic values of MXD3-1 (AUC = 0.906) and MADCAM1 (AUC = 0.812) in the GSE124272 dataset were good. Within the GSE150408 dataset, the following MXD3 biomarkers still had strong diagnostic values: AUC values of 0.676 for MXD3-1 and 0.751 for MXD3-2. We also assessed MXD3-1 (AUC = 0.827) and MADCAM1 (AUC = 0.766), both of which had good sensitivity in TCGA datasets. To predict the incidence of osteoarthritis, Xie et al developed a random forest model and used AUC to screen 7 candidate N6-methyladenosine regulatory factors (IGFBP3, WTAP, IGFBP1, HNRNPC, RBM15B, YTHDC1, and METTL3).^[38]^ Meanwhile, in a study of susceptibility modules and hub genes associated with diabetes mellitus and fracture healing, Ding et al found in a LASSO regression analysis of upregulated key genes that SRPK1, ACSL1, and BCL6 were eventually included in the model. On the other hand, the model of downregulated key genes consisted of HNRPA1P4, SKAP1, ATP6V0E2, and C6orf48. The ROC analyses suggested that both the upregulated and downregulated key gene models demonstrated a satisfactory ability to distinguish patients from the normal population, with AUCs of 0.82 and 0.81, respectively.^[39]^ In this study, the ROC analysis found that the AUC of MXD3-1 was 0.906 in GSE 124272 and 0.827 in CTGA, and the AUC of MXD3 was 0.628 in GSE70768. Therefore, MXD3 has good predictive properties for hip pain and PRAD.

According to the PPI network, 10 distinct genes were shown to have strong associations with MXD3. A larger circle displayed a greater number of nodes for each gene. An association between MXD3 and MXI was established in both the hip pain and PRAD groups. Multiple lines of evidence have pointed to the role of MXI1 as a potential growth suppressor in the prostate. The transfection of complete chromosome 10 into PC3 PRAD cells resulted in a reduction in the tumorigenicity of these cells,^[40]^ suggesting that growth inhibitory genes are present on chromosome 10.^[41,42]^ In the past, we and other researchers discovered that the human MXI1 gene was situated on chromosome 10q24-q25.^[43,44]^ Approximately 30% to 50% of human prostate tumors were found to have deletions that led to the loss of alleles in this particular area of chromosome 10.^[45–47]^ In addition, it was discovered that certain primary human prostate tumors contained mutations in the MXI1 coding sequence that rendered the function of the protein inactive.^[48]^ In conclusion, mxi1-knockout animals that exhibited a tumorigenic phenotype revealed hyperplasia of the prostate.^[49,50]^ It was reported that MXI1 has a role in normal prostate development and may also play a role in human prostate neoplasia based on the increased proliferation of prostatic epithelium in mice lacking MXI1. Because of this, MXD3 and MXI1 might play a significant role in the simultaneous treatment of hip pain and PRAD.

To further examine the roles of MXD3 and MXI1 in tumors, we proved that the expression of MXD3 in tumor tissue was higher than that in adjacent tissues, while the expression of MXI1 in tumor tissue was lower than that in adjacent tissues. These results show that the higher expression of MXD3 and the lower expression of MXI1 are closely related to the occurrence and development of tumors.

Neutrophils, T-helper cells, mast cells, and T cells were shown to play significant roles in the etiology of hip pain and PRAD, according to the findings of the immune infiltration study. Bioinformatics tools were utilized by Gao et al to investigate the close genetic connection between periodontitis and IgAN infections,^[20]^ and they found this association using inverse variance weighting methods. Gungabeesoon et al found that the loss of interferon-responsive transcription factor interferon regulatory factor 1 in neutrophils leads to the failure of antitumor immunotherapy. Neutrophil response depends on the key components of antitumor immunity, including BATF3-dependent dendritic cells, IL-12, and IFNγ. In addition, they found that the systemic neutrophil response induced by antitumor therapy is positively correlated with the prognosis of lung cancer patients. Therefore, neutrophil status may play an important role in mediating effective cancer treatment.^[51]^ Mast cells are immune cells distributed in different parts of the body. Studies have reported their important role in the pathogenesis of neuropathic pain. When stimulated by nerve injury, they release active mediators, mainly histamine and serotonin. The mast cell stabilizer has the effect of relieving pain by preventing mast cells from degranulation. They demonstrate pain relief by weakening the effects of histamine and serotonin.^[52]^ Kruse et al found that effector T cells cluster at tumor-invasive margins. They showed that T-helper type 1 cell-directed T cells and innate immune stimulation reprogram the tumor-associated myeloid cell network toward interferon-activated antigen presenting cells. Meanwhile, T cells and tumoricidal myeloid cells orchestrate the induction of remote inflammatory cell death, which indirectly eradicates interferon-unresponsive and major histocompatibility complex-deficient tumors.^[53]^ Therefore, we speculate that the above cells may play a role in the development of hip pain and PRAD by controlling the activity of immune cells.

Research on single-cell sequencing and single-cell data analysis has gained popularity recently, and these have been applied to numerous tumor studies.^[54,55]^ They can be used to detect the tumor and immunological microenvironment, tumor heterogeneity, and the mechanisms behind tumor formation and evolution.^[56]^ For instance, scRNA-seq in breast cancer can map the tumor microenvironment in the disease by analyzing the multi-omic characteristics of individual cells. This facilitates targeted treatment. To find potential immunotherapeutic targets for gliomas, researchers might look at the functional, molecular, and geographical heterogeneity of tumor-associated immune cells.^[57]^ MXD3 encodes a member of the Myc superfamily of basic helix-loop-helix leucine zipper transcriptional regulators. The encoded protein forms a heterodimer with the cofactor MAX, which binds specific E-box DNA motifs in the promoters of the target genes and regulates their transcription.^[29]^ We found that the MXD3 messenger (m)RNA expression in various normal human tissues, including the immune, internal, nervous system, secretory, muscle, and pancreas, is <10. We also analyzed GSM8172655. We found that it is common for MXD3 to be underexpressed in a variety of cells in single-cell analysis. Mxi1 belongs to the Mad family of proteins, which function as potent antagonists of Myc oncoproteins.^[58]^ This antagonism relates partly to their ability to compete with Myc for the protein Max and for consensus DNA-binding sites and to recruit transcriptional co-repressors.^[49]^ As an effector protein, MXI1 may show a higher expression level than MXD3, which is mainly responsible for encoding transcription factor regulatory protein expression. This demonstrates the unique role and importance of MXI1 in biological processes.

Our study has several strengths. Initially, we employed an intricate and thorough bioinformatics research method as a novel technique for comprehending the connection between the 2 illnesses. Prediction accuracy is increased through external dataset validation. Nevertheless, our work also has shortcomings. Our conclusions were not verified in a single patient and instead depended on several patient cohorts. To confirm any potential future relationships between the 2 disorders, a model of the combination of hip pain and PRAD needs to be constructed. Furthermore, our study did not consider information on the comorbidities, medication, age, or sex of the individuals in the samples, which may account for the results.

## 5. Conclusion

This was the first study to investigate the close genetic link between hip pain and PRAD using bioinformatics technologies. The 2 most significant genes involved in the crosstalk between PRAD and hip pain were MXD3 and MXI1. Immunological responses were triggered by neutrophils and T-helper cells, Tgd cells, and mast cells in the relationship between PRAD and hip pain.

## Acknowledgments

The authors thank TCGA and GEO database for providing data sharing, as well as other online data analysis.

## Author contributions

**Conceptualization:** Liang Huang.

**Data curation:** Yu Xie.

**Formal analysis:** Shusuan Jiang.

**Investigation:** Binbin Gong.

**Software:** Yao Feng.

**Visualization:** Yao Feng.

**Writing – review & editing:** Hong Shan.

## Supplementary Material


