# Supplementary material for: Identification of the shared gene MXD3 signatures and biological mechanism in patients with hip pain and prostate cancer
Source: Medicine (Baltimore). 2024 Sep 13;103(37):e39592. doi: 10.1097/MD.0000000000039592 (PMC11404923; doi:10.1097/MD.0000000000039592)
Supplement: Supplementary file 1 [file medi-103-e39592-s001.pdf]

Supplementary material

Supplementary Table 1. Clinicopathological information of the PRAD patients sets in this study.

Supplemental Figure 1. A heatmap of the DEGs in GSE124272.

Supplemental Figure 2. A heatmap of the DEGs in TCGA.

Supplemental Figure 3. An example dendrogram of WCGNA genes with differential expression. Sample dendrograms in GSE150408 (A) and TCGA (B) are presented.

Supplemental Figure 4. Single sample GSEA (ssGSEA) analysis showing the relative pathways involved with MXD3.

(A-B) ssGSEA analysis showing the relative signature in low back pain patients in GSE150408 (A) and GSE124272 (B).

(C-D) ssGSEA analysis showing the relative signature in PRAD patients in TCGA (C) and GSE70768 (D).

Supplemental Figure 5. Expression pattern validation and diagnostic value.

(A) ROC curve of MADCAM1, MXD3 in GSE150408.

(B) ROC curve of the shared diagnostic genes in GSE70768.

Supplementary Material

Supplementary Table1 | Clinicopathological information of the PRAD patient sets in this study.

**Baseline characteristics**	**Value***
**Age (years)**	54.6 (44-66)
Gender	
Male	11 (100)
**Smoking status**	
Yes	4 (36)
No	7 (64)
**Alcohol history**	

Yes	7 (64)
No	4 (73)
**Grade**	
G1	5 (45)
G2	3 (27)
G3	2 (18)
G4	1 (10)
GX	0
**Stage**	
I-II	6 (55)
III-IV	3 (27)
unknow	2 (18)

***values are expressed as median (range) or n (%)**

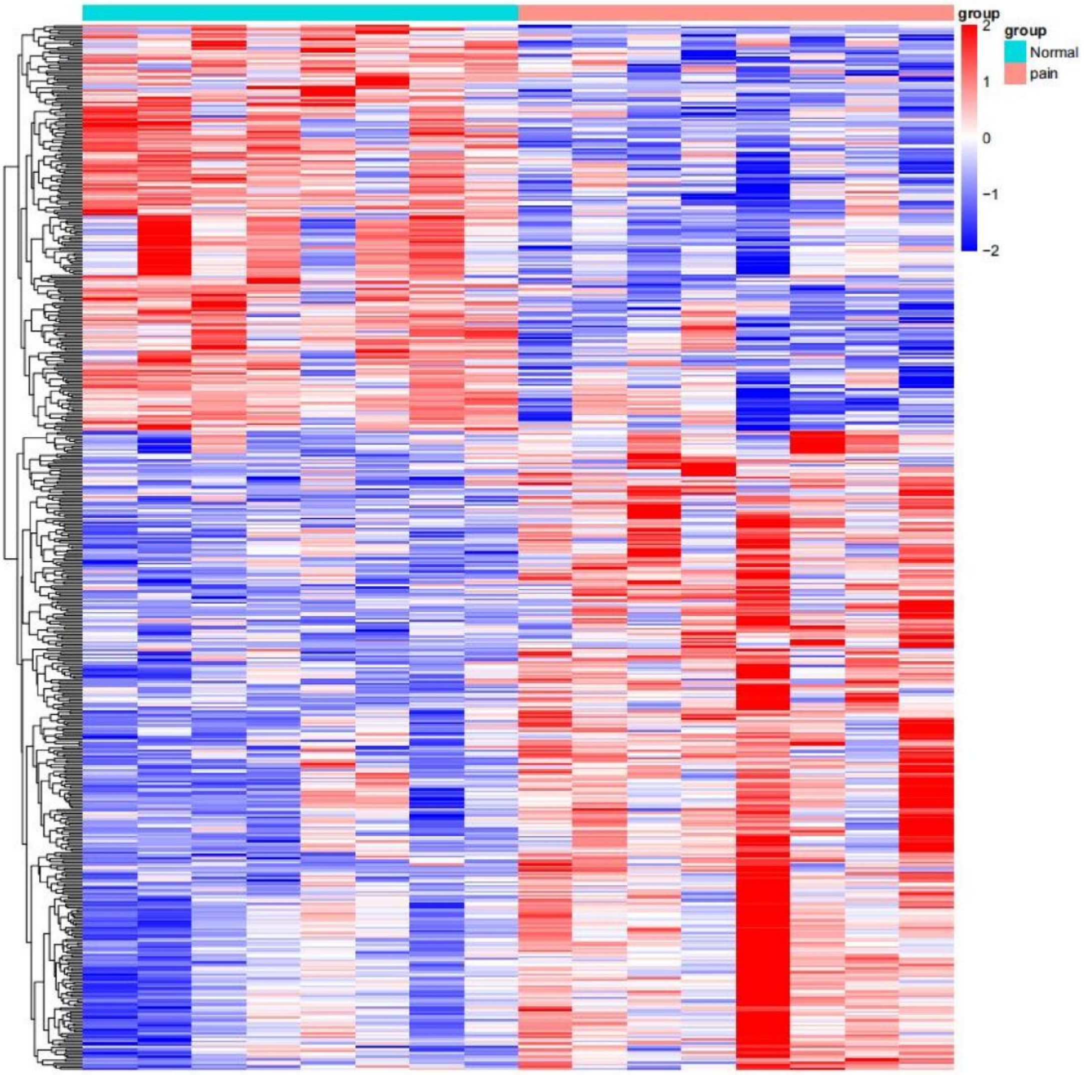
group
2
1
0
−1
−2
group
Normal
pain

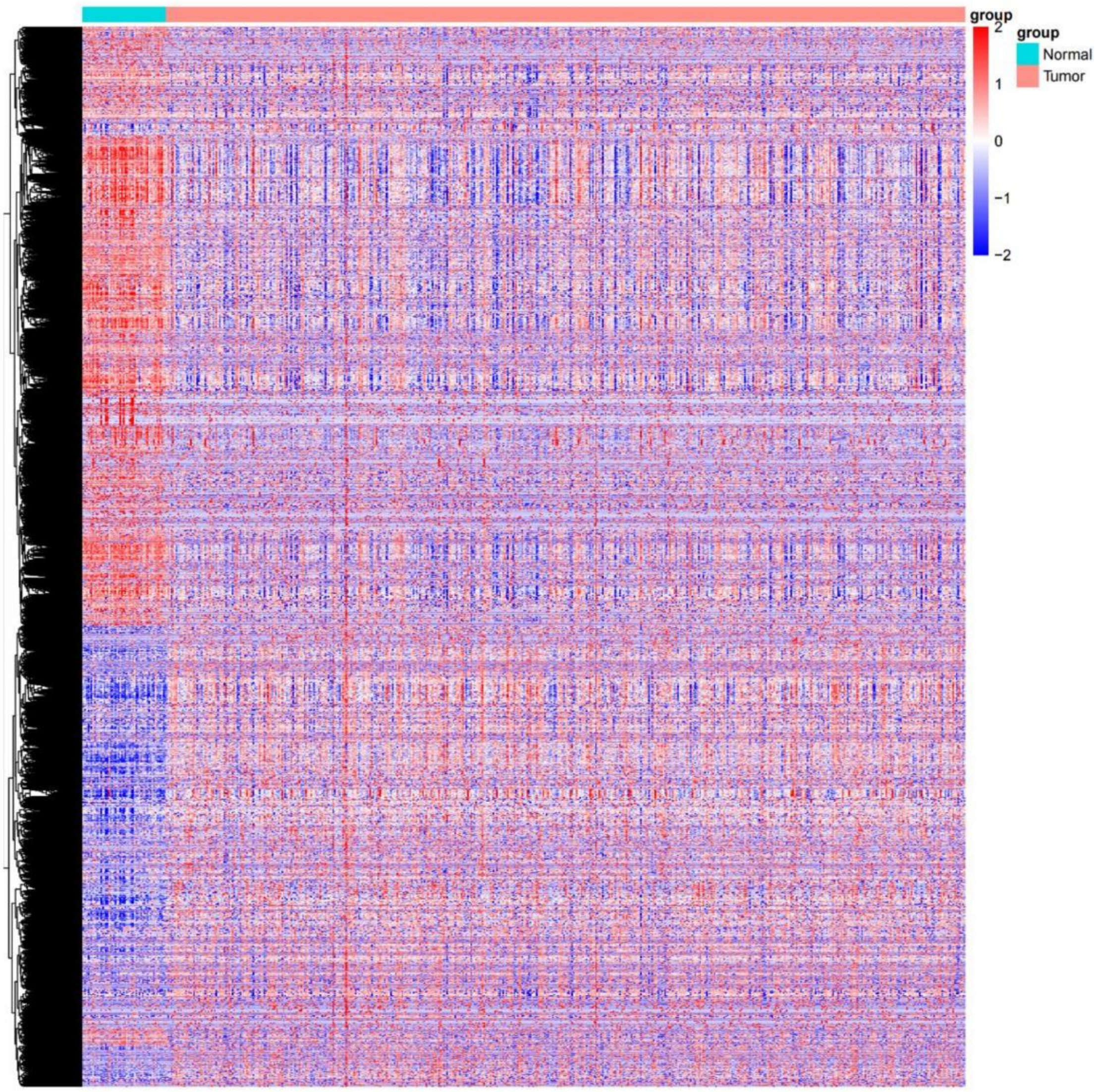
group
2
1
0
−1
−2
group
Normal
Tumor

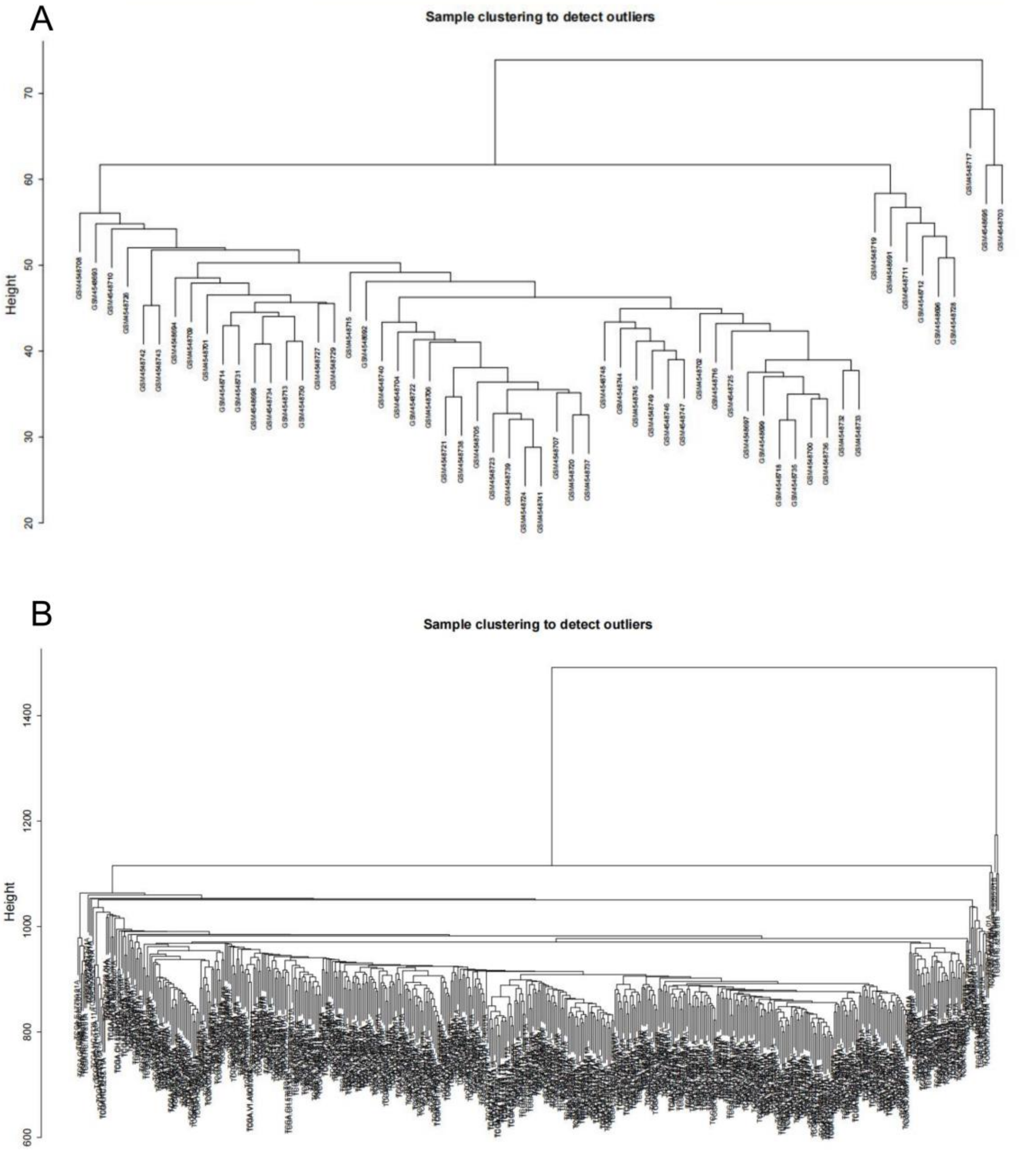
A
Sample clustering to detect outliers
Height
70
60
50
40
30
20
B
Sample clustering to detect outliers
Height
1400
1200
1000
800
600

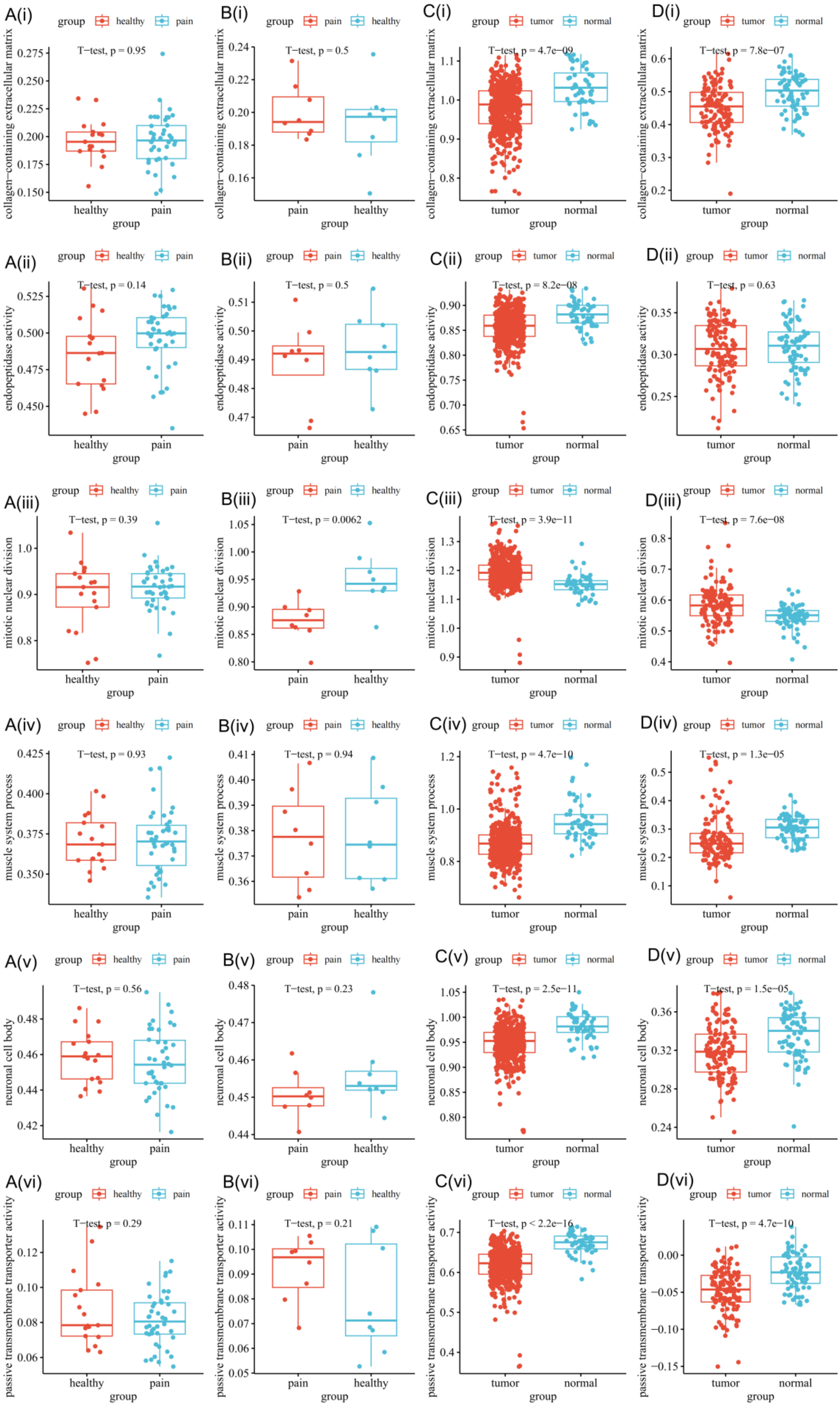
A(i)
group healthy pain
T−test, p = 0.95
collagen−containing extracellular matrix
healthy pain
group
B(i)
group pain healthy
T−test, p = 0.5
C(i)
group tumor normal
T−test, p = 4.7e−09
D(i)
T−test, p = 7.8e−07
A(ii)
T−test, p = 0.14
endopeptidase activity
B(ii)
T−test, p = 0.5
C(ii)
T−test, p = 8.2e−08
D(ii)
T−test, p = 0.63
A(iii)
T−test, p = 0.39
mitotic nuclear division
B(iii)
T−test, p = 0.0062
C(iii)
T−test, p = 3.9e−11
D(iii)
T−test, p = 7.6e−08
A(iv)
T−test, p = 0.93
muscle system process
B(iv)
T−test, p = 0.94
C(iv)
T−test, p = 4.7e−10
D(iv)
T−test, p = 1.3e−05
A(v)
T−test, p = 0.56
neuronal cell body
B(v)
T−test, p = 0.23
C(v)
T−test, p = 2.5e−11
D(v)
T−test, p = 1.5e−05
A(vi)
T−test, p = 0.29
passive transmembrane transporter activity
B(vi)
T−test, p = 0.21
C(vi)
T−test, p < 2.2e−16
D(vi)
T−test, p = 4.7e−10

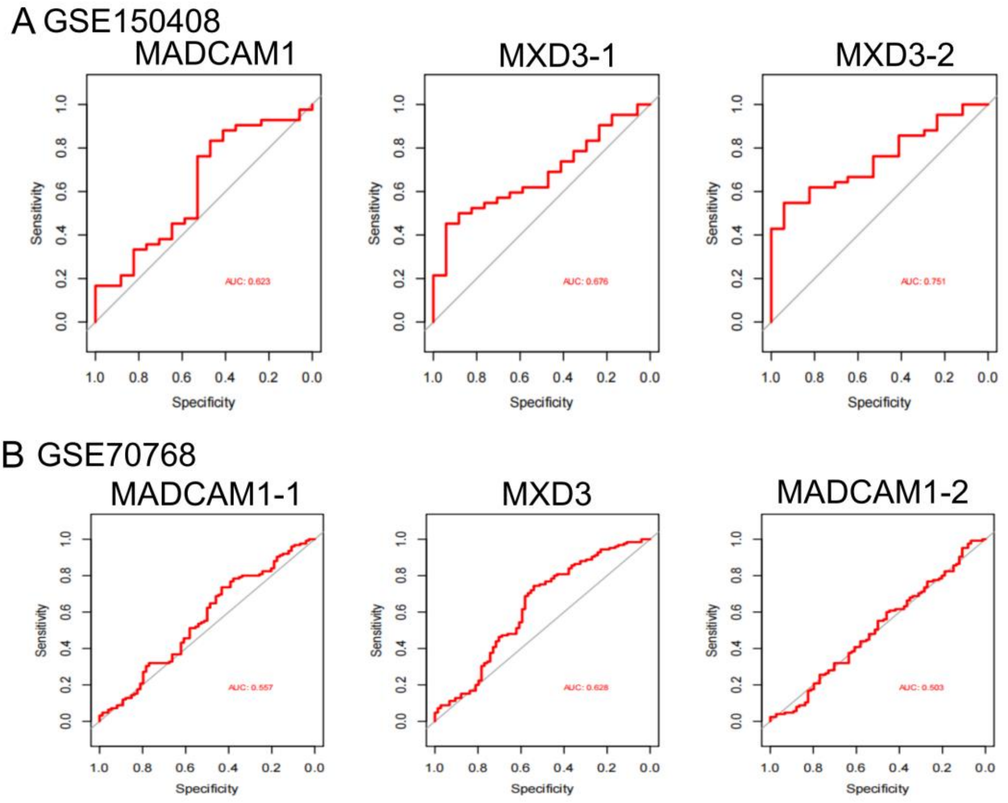
A GSE150408
MADCAM1
AUC: 0.623
MXD3-1
AUC: 0.676
MXD3-2
AUC: 0.751
B GSE70768
MADCAM1-1
AUC: 0.557
MXD3
AUC: 0.628
MADCAM1-2
AUC: 0.503
Sensitivity
Specificity
